# Propagation of tau and α-synuclein in the brain: therapeutic potential of the glymphatic system

**DOI:** 10.1186/s40035-022-00293-2

**Published:** 2022-03-21

**Authors:** Douglas M. Lopes, Sophie K. Llewellyn, Ian F. Harrison

**Affiliations:** Division of Medicine, Department of Imaging, Centre for Advanced Biomedical Imaging, Faculty of Medical Sciences, University College London, Paul O’Gorman Building, 72 Huntley Street, London, WC1E 6DD UK

**Keywords:** Glymphatic, Propagation, Tauopathy, Synucleinopathy, Clearance, Aquaporin-4

## Abstract

Many neurodegenerative diseases, including Alzheimer’s disease and Parkinson’s disease, are characterised by the accumulation of misfolded protein deposits in the brain, leading to a progressive destabilisation of the neuronal network and neuronal death. Among the proteins that can abnormally accumulate are tau and α-synuclein, which can propagate in a prion-like manner and which upon aggregation, represent the most common intracellular proteinaceous lesions associated with neurodegeneration. For years it was thought that these intracellular proteins and their accumulation had no immediate relationship with extracellular homeostasis pathways such as the glymphatic clearance system; however, mounting evidence has now suggested that this is not the case. The involvement of the glymphatic system in neurodegenerative disease is yet to be fully defined; however, it is becoming increasingly clear that this pathway contributes to parenchymal solute clearance. Importantly, recent data show that proteins prone to intracellular accumulation are subject to glymphatic clearance, suggesting that this system plays a key role in many neurological disorders. In this review, we provide a background on the biology of tau and α-synuclein and discuss the latest findings on the cell-to-cell propagation mechanisms of these proteins. Importantly, we discuss recent data demonstrating that manipulation of the glymphatic system may have the potential to alleviate and reduce pathogenic accumulation of propagation-prone intracellular cytotoxic proteins. Furthermore, we will allude to the latest potential therapeutic opportunities targeting the glymphatic system that might have an impact as disease modifiers in neurodegenerative diseases.

## Introduction and background

Ageing is an irreversible process which is associated with neuronal degeneration. According to the current United Nations’ report, there are over 700 million people aged 65 or older around the world, a number that is projected to steadily increase in the next few decades and predicted to reach approximately 1.5 billion by 2050 [1]. Ageing leads to physical deterioration, representing the main risk factor for most neurodegenerative diseases, including Alzheimer’s disease (AD) and Parkinson’s disease (PD) [2, 3]. Indeed, the increase in population growth and ageing is accompanied by a sharp rise in the number of people living with neurodegenerative diseases. Studies estimate that there are 40–50 million people living with AD and other dementias around the world, and in 2016 it was reported that dementia alone was the fifth leading cause of death globally, accounting for over 2 million deaths worldwide per year [1]. Given their nature and prevalence, neurodegenerative diseases represent a major financial burden to society, and one of the most pressing global health challenges of our time.

Neurodegenerative diseases are largely associated with a progressive destabilisation of the neuronal network, disturbance in synaptic transmission, synaptic loss and changes in intracellular signalling, which ultimately lead to neuronal death [4–6]. Although the hallmarks of most neurodegenerative diseases have been described, very little is known about the cellular processes underlying their progression, making the development of new therapeutic targets extremely challenging. With virtually no successful treatments available to patients, neurodegenerative diseases are therefore one of the largest unmet medical needs today [7]. Understanding the mechanisms that trigger and underlie these disorders is therefore crucial and urgently needed for the development of new therapeutic approaches.

### Amyloids and neurodegenerative disease

Most common neurodegenerative diseases are characterised by hallmark accumulation of protein deposits either inside or outside cells in the brain. These intra/extracellular accumulations are composed largely of a particular protein as its major component, the identity of which varies between neurodegenerative disease pathologies. Many of these proteins can enter an ‘amyloid state’, in which they form elongated fibres with spines consisting of many-stranded β-sheets which run in parallel with the filament axis [8, 9]. Among the most studied proteins in this group are amyloid-β plaques in AD; tau neurofibrillary tangles in frontotemporal dementia with parkinsonism, and AD; α-synuclein Lewy bodies and neurites in PD, and dementia with Lewy bodies; huntingtin inclusions in Huntington’s disease; TDP-43 inclusions in amyotrophic lateral sclerosis; and Prion protein (PrP) inclusions in prion disease (spongiform encephalopathies) [8, 10].

**Fig. 1:**
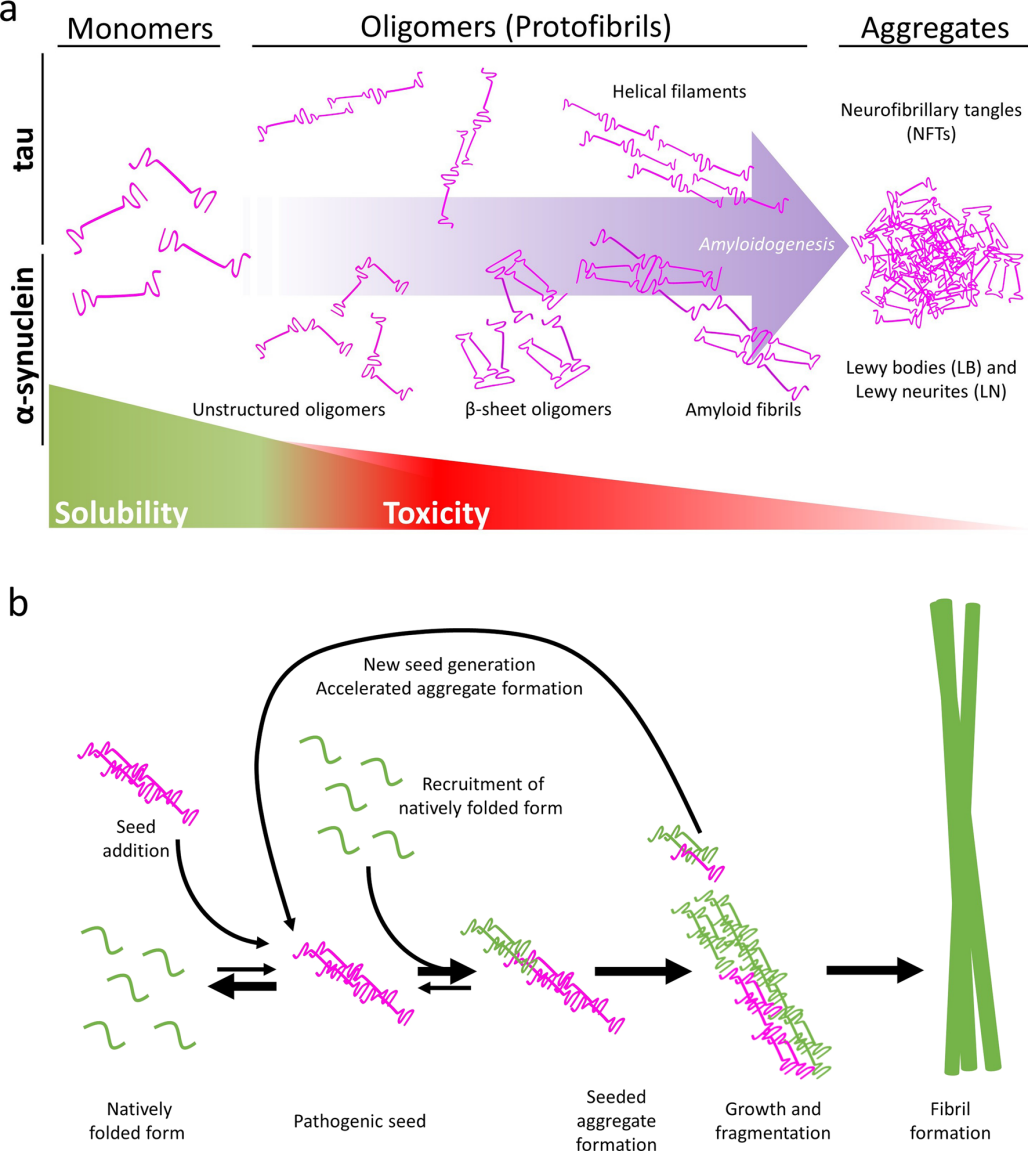
The formation of cytotoxic species of tau and α-synuclein leading to formation of insoluble filaments. **a** Monomeric forms of these proteins, which are notably soluble, are capable of recruiting and binding to similar structures, giving rise to oligomeric configurations and more complex structures—helical filaments in the case of tau and amyloid fibrils in α-synuclein protein. This process leads to the formation of insoluble aggregates (tau neurofibrillary tangles and α-synuclein Lewy bodies and neurites) which can accumulate intracellularly and lead to cell death. **b** As indicated by the arrows, the formation of a pathological seed is an energetically unfavourable event, and as such is rare. Once a seed has formed however, monomers of the protein in its natively folded form can change shape and join the initially formed seed in creation of a seeded aggregate. Fragmentation of this aggregate generates new seed forms, accelerating the formation of further aggregates. Recruitment of further monomers/oligomers results in its growth and formation of fibrils (Adapted from Goedert [97])

These proteins have a unique property of acting as a template for their own replication: beginning with a slow nucleation phase which, through a series of intermediate states (Fig. 1a), forms the initial segment of the amyloid spine (Fig. 1b) [8, 11]. Protein monomers or oligomers can then bind to the ends of the initial structure by conformational conversion [8], and as the fibril grows, depending on its conformational stability, it can break, hence inducing a self-propagating pathway of generation and release of new amyloid ‘seeds’ (Fig. 1b). These amyloid structures are extremely stable and, in the case of neurodegenerative disease-associated proteins, are thought to result in neurotoxicity. This can occur through the disruption of cellular function by either a toxic gain-of-function effect, and/or by molecular sequestration, leaving cells starved of molecules required for their function [12].

Although the identity of the initial trigger for amyloid assembly in neurodegenerative disease is unknown, several* in vivo* conditions that promote the energetically unfavourable amyloid formation have been implicated in disease aetiologies. For example, mutations that destabilise the native form of the protein; increased concentrations of amyloid-prone proteins or de novo generation of amyloid species through native protein cleavage [8]. Notably, dominantly inherited neurodegenerative diseases are often associated with mutations in the gene encoding the protein that forms amyloid structures, such as mutations in *MAPT*, the tau gene, in inherited forms of frontotemporal dementia with parkinsonism; and mutations in *SNCA*, the α-synuclein gene, in familial forms of PD. Although many proteins associated with neurodegenerative diseases are capable of forming amyloid aggregates, in this review we will be focusing on intracellular proteins, namely, the two most prevalent proteins tau and α-synuclein, which are thought to drive neurodegeneration, yet their biological relationship with the glymphatic system is only starting to be explored.

### ‘Prion-like’ propagation

In prion disease, the accumulation of amyloid aggregates of PrP throughout the brain leads to gross neurodegeneration and cerebral atrophy. However, what is most commonly associated with this neurodegenerative disorder is its transmissibility. The infectious protein ‘seed’, or ‘proteinaceous infectious particle’ (abbreviated to ‘prion’ by Stanley Prusiner in his seminal 1982 work [13]) can transmit between individuals, and cause its own amplification and replication by recruiting the endogenously expressed PrP^C^ protein in the brain into the pathological PrP^Sc^ form, inducing pathological amyloid aggregation and neuronal toxicity [14]. Indeed, this seeded amplified aggregation of PrP can be replicated* in vitro*, by incubating minute amounts of PrP^Sc^ ‘seeds’ with excess concentrations of PrP^C^ [15]. Although prion disease is classically considered as an infectious disease, 99% of cases are sporadic or genetic in origin [16], raising the intriguing suggestion that the trigger of seeded accumulation in the brain can also begin endogenously.

There is limited evidence indicating that the more common neurodegenerative diseases, such as AD and PD, are transmissible between organisms [17], yet they too present with amyloid state aggregates composed of different proteins in the brain, progressive neurodegeneration, and sporadic as well as genetic origins. With that in mind, given the parallels between the amyloid state accumulations in prion disease and these other neurodegenerative disorders, rigorous research efforts have been placed in the last decade on the possible ‘prion-like’ action of other amyloid-prone protein species in the brain, a term first used by Adriano Aguzzi in 2009 [18]. Notably, tau and α-synuclein have demonstrated significant degrees of prion-like action in various experimental settings, as we discuss later [19–23].

**Fig. 2:**
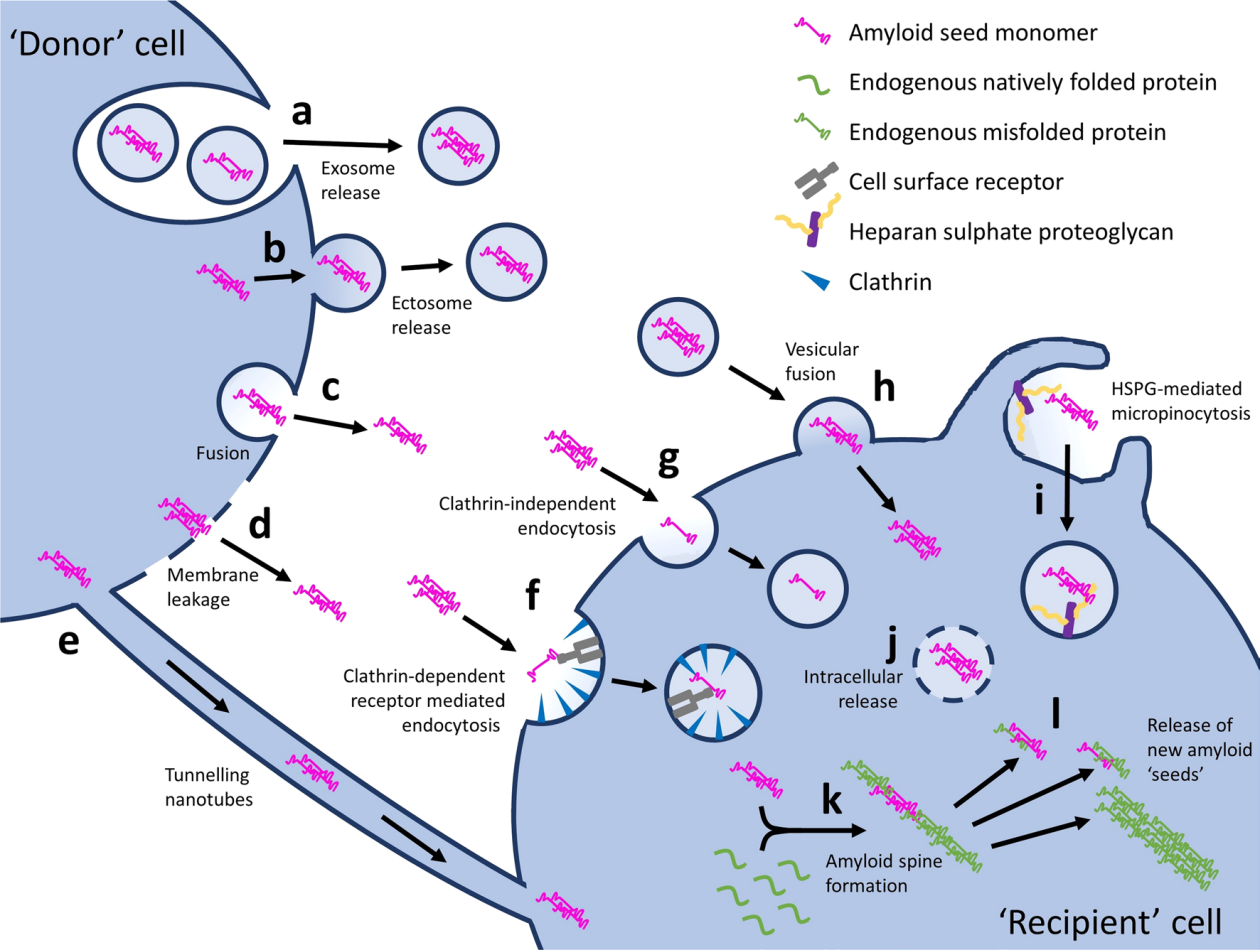
Cell-to-cell spread of prion-like protein species. Prions are proposed to be spread from ‘donor’ to ‘recipient’ cells by numerous mechanisms. ‘Naked’ prions can be released into the extracellular space through either exosomal release or leakage through damaged cell membranes (**c** and **d**, respectively), where they can, in turn, become internalised by mechanisms such as direct pinocytosis, a process thought to occur *via* both clathrin-dependent receptor mediated endocytosis (**f**) and clathrin-independent endocytosis (**g**), resulting in intracellular release (**j**). Heparan sulphate proteoglycans (HSPGs), transmembrane and lipid-anchored cell surface receptors that interact with a variety of ligands, have also been shown to mediate internalisation of prions, *via* HSPG-mediated micropinocytosis (**i**): an endocytic process characterized by binding of the prion ligand to surface-bound HSPG, actin-driven membrane ruffling, internalization of extracellular fluids, and formation of large intracellular vacuoles. Prions can also be released within exosomes (**a**) or ectosomes (**b**), which can be internalised through vesicular fusion (**h**). Tunnelling nanotubes, F-actin containing membranous bridges which connect the cytoplasm of remote cells with one another, can also facilitate cell-to-cell exchange (**e**). Ultimately, once inside the new host cell, prion-like amyloid-prone proteins are thought to initiate amyloid assembly, through recruitment of endogenous natively folded protein monomers or oligomers leading to formation of an initial segment of an amyloid spine (**k**). This spine can then break, inducing further intracellular propagation of amyloid formation and release of new amyloid ‘seeds’ (**l**), which can then be released and propagate to new unaffected cells by the aforementioned cell-to-cell translocation pathways

The ‘prion’ hypothesis not only encompasses the ability of the prion agent to self-propagate and induce release of further prions, but also the cell-to-cell transmission and spread throughout the brain. In the past decade, a broad consensus on the fundamental cellular events that underlie the transcellular propagation of prion-like agents in the brain was reached [24]. Namely, that prion-like ‘seeds’ can move between cells, allowing them to become internalised by ‘recipient’ cells where they trigger further intracellular aggregation involving recruitment of endogenous natively folded protein molecules to form amyloid aggregates (Fig. 2). There is still debate in the literature as to how translatable this model of transcellular propagation and spread of pathological protein species is to the clinical scenario [25]. Nonetheless, as there appears to be a clear link between proteinaceous propagation and clinical symptoms in neurodegenerative diseases, understanding the mechanisms of cell-to-cell translocation of pathological prion-like protein species in the brain may lead to identification of novel therapeutic targets for numerous neurodegenerative diseases.

Prions are proposed to be released by affected ‘donor’ cells through several different mechanisms, the simplest being direct release into the extracellular space through either exosomal release (Fig. 2c) or leakage through damaged cell membranes (Fig. 2d). Indeed, studies have shown that treatment of neurons with compounds that interfere with exosome release mechanisms result in significant changes in the extent of extracellular release of amyloid-prone protein species [26, 27]. Further, release of protein species can also be achieved through passive leakage across the damaged plasma membrane, a process exacerbated by the presence of fibrillar protein constructs themselves, e.g. fibrillar α-synuclein [28, 29]. These ‘naked’ prion constructs in the extracellular space, in turn, become internalised through numerous mechanisms, including direct pinocytosis, a process thought to occur both clathrin-dependently (Fig. 2f) and independently (Fig. 2g) [30], and attributable to monomer endocytosis only [31]. Oligomers and short fibrils, on the other hand, can be internalised through receptor-mediated endocytosis (Fig. 2f) [32]. For example, lymphocyte-activation gene 3 (LAG3) has been proposed to mediate the internalisation of α-synuclein [33], which is then released intracellularly (Fig. 2j). Recent studies, however, have disputed the role of LAG3 in α-synuclein internalisation in neurons, as it has been demonstrated that this gene is not expressed in neuronal cells, but only in microglia and macrophages [34]. Similarly, heparan sulphate proteoglycans (HSPGs), transmembrane and lipid-anchored cell-surface receptors that interact with a variety of ligands, have also been shown to mediate the internalisation of prions [35, 36]. This process is known as HSPG-mediated micropinocytosis (Fig. 2i), an endocytic process characterized by binding of the prion ligand to surface-bound HSPG, actin-driven membrane ruffling, internalization of extracellular fluids, and formation of large intracellular vacuoles [35]. Prion structures can also be released into the extracellular space as either exosomes [37] (Fig. 2a) or ectosomes [38] (Fig. 2b), which can in turn be internalised by ‘recipient’ cells through vesicular fusion (Fig. 2h). Lastly, protein species have also been shown to be exchanged directly between ‘donor’ and ‘recipient’ cells *via* tunnelling nanotubes (Fig. 2e), which are F-actin-containing membranous bridges that connect the cytoplasm of remote cells with one another [39, 40]. This phenomenon has been observed to not only occur between neurons, but also between astrocytes, pericytes and also between cell types [40, 41]. Once inside the new host cell, prion-like amyloid-prone proteins can act as templates for their own replication (see Fig. 1b). This process leads to the formation of an initial segment of a new amyloid spine, which, in turn, can break and release new amyloid seeds and propagate to surrounding unaffected cells (Fig. 2k, l).

A number of these mechanisms of transcellular prion propagation involve the release and uptake of pathological seed components into/from the extracellular space, highlighting this part of propagation cascade as a potential therapeutic target. Correspondingly, although the mechanisms that facilitate tau intercellular propagation through secretion and uptake are not fully understood, multiple studies in recent years have taken an immunotherapeutic approach, to aid clearance of extracellular tau and α-synuclein in an attempt to block their propagation throughout the brain [42–45]. Though the precise mechanisms of immunotherapeutic clearance of these proteins from the extracellular space are still not well defined, it has been proposed that antibodies could promote clearance from these spaces, affect brain clearance, or even promote intraneuronal clearance [46].

The concentrations of extracellular proteins such as tau and α-synuclein in the interstitial fluid (ISF) are maintained based on a balance between the extent of cellular release into the space, and extracellular clearance from the space. Tau, for example, has been found to have a particularly low level of extracellular turnover (~ 11–17-day half-life [47]), making it prone to propagation if misfolded in a seed-competent manner. Acceleration of endogenous mechanisms which clear the extracellular ISF of these protein species could therefore potentially hold therapeutic promise against their propagation in the brain. Notably, the glymphatic pathway has been shown to have a critical influence on elimination of extracellular molecules, such as amyloid-β, tau and α-synuclein [48–52]. This pathway may thus have novel therapeutic potential against prion-like propagation in the raft of neurodegenerative disease states.

### The glymphatic system

Studies proposing the existence of a system in the brain, equivalent to the peripheral lymphatic system which drains fluid and clears out waste, date back centuries [53]. Anatomists Rudolf Virchow and Charles Robin were among the pioneers to describe the existence of spaces around blood vessels, including in the brain, in the 1850s, which are now referred to as components of a perivascular system composed of ‘Virchow-Robin’ spaces [54]. Today, the term ‘perivascular space’ is widely accepted to describe the space surrounding the blood vessels which can communicate with the subarachnoid space, where CSF circulates [55]—though the communication between these two spaces is still disputed by some authors [56, 57]. Shortly after the initial descriptions of the perivascular spaces in the brain, in 1869, Gustav Schwalbe provided experimental evidence to show that dyes injected into the cranial subarachnoid spaces of different laboratory species could enter the lymphatic vessels and lymph nodes of the head and neck [58]. These experiments provided a clear link between the early observations of Virchow and Robin to the possible existence of a lymphatic drainage-like system in the central nervous system (CNS). Subsequent studies further demonstrated that injection of a dye either into the CSF or directly into the subarachnoid space could enter the perivascular space, suggesting communication between the CSF and the ISF in the brain [54]. Collectively, these studies provided strong evidence pointing to the existence of a fluid-mediated clearance system in the CNS.

**Fig. 3:**
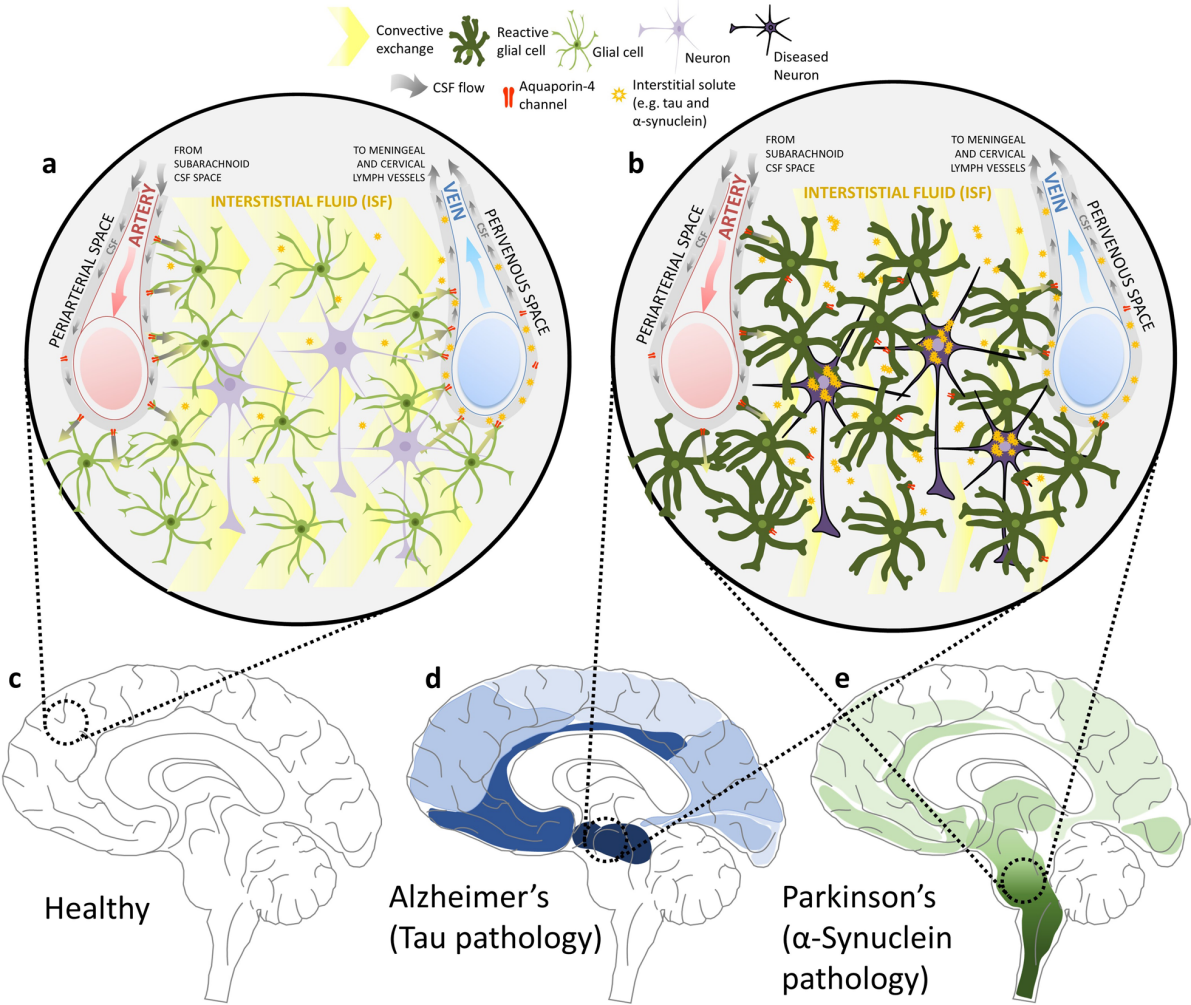
The glymphatic system in neurodegenerative disease. **a** An overall view of the glymphatic system and its main components in health (**c**): the CSF that flows alongside the arteries (from the subarachnoid CSF spaces) moves across the brain parenchyma *via* convective exchange (yellow chevrons). The movement is driven by pressure gradients and the convective transport is facilitated by a network of glial projections (green cells) and aquaporin-4 channels expressed on their endfeet (red structures). As the CSF passes through the brain parenchyma, it carries the interstitial solutes present in that space towards the perivenous space, from where it gets cleared out of the brain towards meningeal and cervical lymph vessels. **b** Failure of the glymphatic system in neurodegenerative disease (e.g. **d** and **e**): aquaporin-4 becomes depolarised from astrocytic endfeet, leading to reduced convective exchange of paravascular CSF with interstitial fluid. This leads to reduced parenchymal clearance of proteins such as tau (**d** depicted in blue in the advanced Braak stage Alzheimer’s scenario) and α-synuclein (**e** depicted in green in the advanced Braak stage Parkinson’s), facilitating their cell-to-cell propagation throughout the brain

The glymphatic system was first described by Maiken Nedergaard and colleagues in 2012, who proposed the existence of a system that relies on the interplay of three main components: the CSF influx route, the ISF clearance route, and the trans-parenchymal exchange pathway, which depend upon astroglial cells (Fig. 3a, c) [49, 59, 60]. By infusing fluorescent tracers into the brain cisterns of anaesthetised mice, this breakthrough study tracked the movement of CSF and demonstrated that after infusion, CSF tracers quickly entered the brain parenchyma in a paravascular fashion, spreading throughout the whole brain [49, 59]. Importantly, the study showed that CSF tracers that exchanged into the interstitial space were rapidly cleared away again, reflecting the existence of a pathway which allows the exchange of CSF and ISF, thus providing a communicative link between these two fluid systems [49]. These observations were pioneering in describing the existence of a brain-wide pathway for fluid transport in mice which resembles the peripheral lymphatic system, demonstrating that CSF enters the brain’s interstitium, exchanges with ISF, and is cleared out along large-calibre draining veins (Fig. 3a). This was termed the ‘glymphatic system’, based on its functional homology with the peripheral lymphatic system and its dependence upon glial cells [59, 60].

Astroglial cells are proposed to form a key link between the para-arterial CSF influx and the para-venous ISF efflux pathways of the glymphatic system [49]. According to the initial model put forward in 2012, astrocytes, known to be in close physical contact with the brain vasculature, facilitate the otherwise unfeasible movement of fluid and solutes through the brain parenchyma, from the periarterial to the perivenous spaces [49, 60]. A crucial feature of these specialised supporting glial cells is the expression of the protein aquaporin-4 (AQP4), a water-specific channel present on the astrocytic endfeet which ensheath the brain’s vasculature (Fig. 3a) [60, 61]. Notably, AQP4 is considered the main water channel expressed in the brain, and unlike its choroid plexus-localised counterpart AQP1, it is thought not to play a role in epithelial fast water transport [61]. In their description of the glymphatic system, Iliff et al. proposed that AQP4 is the key channel which allows the directional flow of CSF, which is driven by pressure gradients and convective transport, through a network of glial projections [49]. Indeed, when CSF tracer influx was imaged *ex vivo*, tracer movement into the brain parenchyma was significantly reduced in AQP4-knockout mice compared to wild-type controls [49, 60, 62]. Importantly, by injection of radiolabelled mannitol into the striatum, this study also showed that the rate of fluid and solute clearance from the brain’s interstitial spaces was significantly suppressed in AQP4-knockout animals, further demonstrating the pivotal role of AQP4 in the glymphatic clearance system [49, 60, 62]. Adding to these findings, the study also proposed a crucial role for the glymphatic system in the context of neurodegenerative disease in its clearance of cytotoxic waste products, namely amyloid-β, from the brain [49]. This was followed up by other studies, also implicating the clearance of tau from the brain by this pathway [48, 50], and also α-synuclein [51, 52]. Many additional studies have also shown that the astrocytic AQP4 is essential for fast glymphatic transport in different systems and disease models [48, 62–67], further emphasizing the central role of astroglial cells in this CNS solute clearance system.

Ageing appears to pose a significant risk factor to glymphatic misfunction. Scientific evidence points out that ageing represents multiple threats to the normal function of the glymphatic system, as astrocytic, CSF production and sleeping pattern differences are commonly found in older individuals. One of the pioneering pre-clinical studies looking into the possible changes in glymphatic function during ageing, compared this system among young, middle-aged and old rodents [68]. Results showed a striking decrease in interstitial CSF influx measured using cisternal delivery of fluorescent tracers, with middle-aged animals showing approximately half as much CSF influx compared to young mice, and a reduction of nearly 80% in old compared to young animals [68]. But what could be driving this loss of CSF influx? The same study showed that this change was associated with loss of polarisation of AQP4 in astrocytic endfeet, despite no overall change in AQP4 expression levels, but a marked increase in the number of astrocytes, particularly surrounding larger vessels in the brain [68]. With ageing, AQP4 became localised to astrocytic parenchymal processes rather than endfeet, therefore dysregulating water transport driven by these cells [60, 68]. Notably, the authors observed that certain areas of the brain, such as the striatum and the hippocampus, appeared to have a greater decrease in AQP4 polarisation in older animals [68]. Further, studies in different mammalian species have also found a direct correlation between age and decrease in both production and pressure of CSF within the brain [60, 69–71], reinforcing the hypothesis that glymphatic activity declines with age. In humans, a compelling relationship has also been observed between impairment of the glymphatic pathway and ageing [72]. Using magnetic resonance imaging to evaluate glymphatic flow in the aged, Zhou and colleagues demonstrated an impairment in glymphatic circulation as well as in meningeal lymphatic outflow in older subjects, with the latter appearing to be downstream of the glymphatic pathway [72, 73]. Adding to this, sleep is crucially important for glymphatic clearance, which will be discussed later, and it is well established that sleep quality decreases with age, with insomnia, interrupted and superficial sleep as well as shorter sleep duration being common among older adults, causing a decline in nocturnal brain waste clearance [74]. Although further studies are necessary to understand whether the failure of the glymphatic system in ageing contributes to the accumulation of harmful proteins, whether these protein waste products accelerate the effects of age on the glymphatic system, or if a concomitant, collaborative process is at play, is yet to be established.

It is important to note, however, that the glymphatic system does not work in isolation to remove soluble waste products from the brain; there are various overlapping clearance systems which work in concert to achieve and maintain parenchymal homeostasis (for reviews see [75, 76]). Waste solutes in the brain can be enzymatically degraded, cleared directly into the blood at the blood–brain barrier, or transported into the CSF by any number of converging mechanisms, glymphatic clearance being one. Indeed, since its initial description in 2012, the glymphatic pathway has received much attention and interest, and rekindled research efforts into many of these clearance pathways, CSF dynamics and fluid flow in the brain. It also sparked some controversy [77, 78]. For example, in their examination of perfusion-fixed tissues, Carare and Weller suggest that the perivascular fluid movement takes place along the basement membranes of the smooth muscle cells of the cerebral arteries only, rather than in the perivascular space of both veins and arteries, and antiparallel rather than parallel to vessel blood flow [79]. This has been re-challenged; Mestre et al. [80] suggested that this observation was artefactual as a result of vessel and perivascular space collapse due to perfusion fixation. Further, based on experimental results, Smith et al. [81] questioned and contradicted the then newly proposed glymphatic pathway, arguing that the transport of solutes in the brain parenchyma occurs in a diffusive fashion, rather than by convective transport as proposed by Iliff and colleagues [49]. Importantly, the authors of this study provided evidence to suggest that AQP4 gene deletion does not impair the transport of solutes from the subarachnoid space to the parenchyma in mice or rats, and dismissed the central role of these water channels in solute transport within the brain. These findings have been re-challenged; a recent study, carried out by a consortium of laboratories across the world, re-evaluated the role of AQP4 in the glymphatic clearance pathway [77]. This rebuttal study extensively examined the role of AQP4 in glymphatic transport using five genetically modified AQP4 mouse lines, including the line used by Smith and colleagues. The group concurred that the CSF influx was higher in wild-type mice than in each of the AQP4 knockout lines tested [77]. Importantly, the study highlighted the central role not only of AQP4, but also of its membrane-binding complex, and suggested that invasive procedures, such as intracerebral injections, may themselves disrupt and impair glymphatic system function [77]. In addition, the authors, along with others, propose that experimental variables such as anaesthesia, age, tracer delivery methods and perfusion-fixation could explain the discrepancy in the findings [77, 80], further reinforcing the pivotal role of AQP4 and the perivascular space in the function of the glymphatic system in the rodent brain. Given the controversy surrounding the glymphatic system, in recent years the pioneering lab (led by Maiken Nedergaard) has published opinion articles reviewing the current areas of controversies, debating data and posing questions that remain outstanding regarding the system [78, 82, 83]. More recently, this has been followed up with an exhaustive *Physiological Reviews* paper, which brings together the current state of the field of fluid transport in the brain, providing an all-encompassing review on where the glymphatic system fits in the brain’s physiological mechanisms for fluid influx and efflux and importantly providing in-depth discussion and point-by-point evaluation of the debated areas of contention [76]. Readers should therefore refer to this work for more expansive discussion and explanation of the controversies surrounding this area of brain physiology.

Controversy aside, the glymphatic system of fluid exchange in the brain has been shown to have a critical influence on clearance of the extracellular space and elimination of molecules prone to aggregation in neurodegenerative diseases, namely amyloid [49] and tau [48, 50]. Like tau, α-synuclein also causes neuronal cell death through intracellular aggregation, and the neuron-to-neuron and region-to-region patterns of propagation of these proteins in the brain suggest that the extracellular space acts as an essential transport conduit for their spread [51, 52]. As has been described above, age, and associated changes in neuroinflammation have been shown to alter glymphatic function [68–70, 72]. So too has neurodegeneration, which is described below. In addition, genetic variation, in the form of single nucleotide polymorphisms in the *AQP4* gene which encodes the water channel central to glymphatic function, has been shown to be associated with differences in sleep quality [84], amyloid-β deposition in the brain [85], and altered rates of cognitive decline after AD diagnosis [86]. Taken together, these findings suggest that the glymphatic system may be present as a novel therapeutic target for neurodegenerative pathologies (Fig. 3). Below we will review the evidence showing the propagation-prone nature of these two intracellular proteins in the context of neurodegeneration. Further, we summarise what is known on the involvement of the glymphatic system in their clearance, advocating future studies aimed at therapeutically modulating this system for the treatment of tauopathies and synucleinopathies.

## Tau

Tau is a microtubule-associated protein present in the axons of neurons, where it stabilises microtubule bundles and plays a key role in microtubule assembly, dynamics and spatial organisation of neuronal cells [87, 88]. Although tau is largely recognised for its role in binding and stabilising microtubules in axons, it has recently been proposed that instead, tau protein allows them to be labile [89, 90]. Furthermore, it has also been suggested that tau plays a role in other cell compartments, including regulation of synaptic plasticity at the dendrites and RNA stability in the nucleus [90, 91], which further highlights the importance of this protein for neuronal stability and function.

**Fig. 4:**
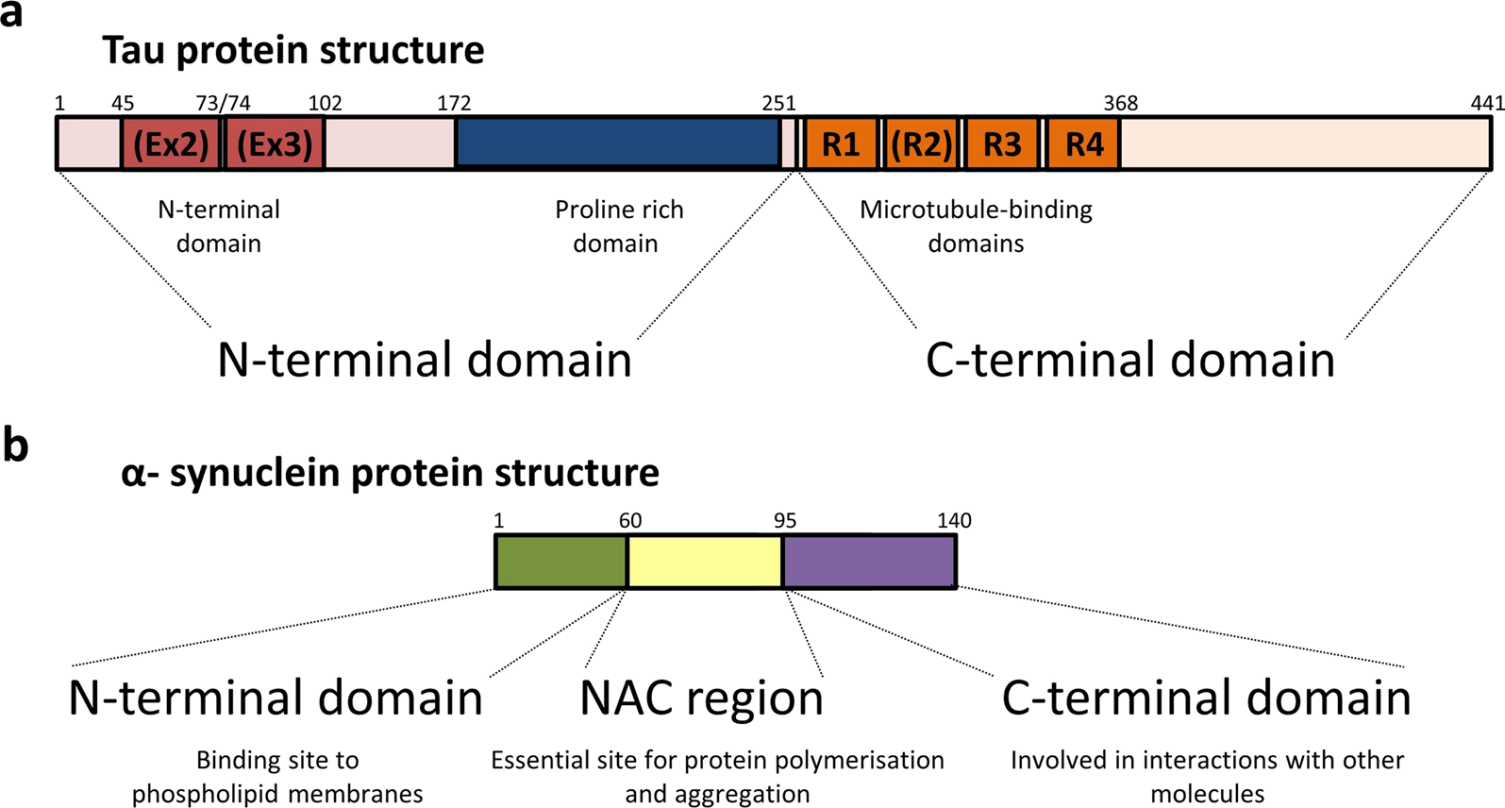
Schematic structure of tau and α-synuclein proteins. **a** Tau protein contains three main domains: an N-terminal domain (red/pink), a proline-rich domain (blue), and a microtubule-binding domain (orange). Tau isoforms can range between 352 and 441 amino acids in length; containing zero, one, or two N-terminal repeats (encoded by exons 2 and 3—shown in dark red), and three or four C-terminal microtubule-binding domains (presence or absence of R2, encoded by exon 10)—which define the 3R and 4R tau species. **b** In contrast**,** α-synuclein is a relatively short protein (140 amino acids in length), but can also be divided into three regions: an N-terminal domain (green), a non-amyloid-component (NAC) region (yellow), and a C-terminal domain (purple), with each of their correspondent functions shown

The tau protein itself is architecturally complex. It is encoded by a single gene, *MAPT*, and in the CNS six different isoforms of the protein exist, ranging from 352 to 441 amino acid residues in length, differing in the presence or absence of exons 2 and 3 (N-terminal domain), and exon 10 (microtubule-binding domain) (Fig. 4a) [87, 88, 90, 92]. The isoforms that carry the second microtubule-binding repeat (encoded by exon 10) result in tau with four repeated microtubule-binding sequences, named 4R tau, whereas the isoforms which lack this second repeat domain have only three, therefore named 3R tau [90]. It has been postulated that in some tauopathic neurodegenerative diseases, the accumulation and proportion of either of these isoforms (3R and 4R) dictates the severity, location, spread and nature of the disease. Given the evidence, it is therefore crucial to consider the composition of tau when studying its link to some disease and model systems. It is also important to note that although there is great similarity between human and mouse tau, with up to 89% homology in some isoforms, the adult human brain contains both 3R and 4R isoforms, whereas only 4R tau is present in adult mice [90]. These differences may limit the success of murine genetic models to study certain aspects of tau pathologies, which will be discussed later.

Tau protein is linked to neurodegenerative diseases. Tau is normally highly soluble, without a well-defined secondary or tertiary structure [93, 94]. However, under certain conditions, tau assembles into β-sheet–rich insoluble amyloid fibrils, commonly known as paired helical filaments, to form neurofibrillary tangles (NFTs) (Fig. 1a) that are highly toxic to neurons [93, 94]. In addition, tau protein can go through several different post-translational modifications, including acetylation, deamination, methylation, demethylation and phosphorylation [95]. Indeed, phosphorylation is reported to decrease the affinity of tau for microtubules, and under pathological conditions, hyperphosphorylation of tau protein prevents its binding to microtubules, resulting also in its accumulation in the cytosol and consequent formation of intracellular NFTs [87, 90]. Disorders in which these changes in tau structure and conformation occur, leading to its intracellular aggregation, are commonly referred to as tauopathies.

The term tauopathy was first used by Ghetti and colleagues when describing an autosomal dominant familial disease characterised by abundant fibrillary deposits of tau protein in neuronal and glial cells [96]. Following this report, key studies identified mutations in *MAPT*, and linked them to familial forms of frontotemporal dementia with parkinsonism [88, 97]. Since then, the term tauopathy has been used for all sporadic or familial neurodegenerative disorders presented with filamentous accumulations of hyperphosphorylated tau protein in neurons, microglia and astrocytes [90, 98]. To date, over 20 different neurodegenerative diseases have been reported to be associated with tau accumulation, including AD, parkinsonism-dementia complex, and chronic traumatic encephalopathy [88].

### Tau propagation

The first evidence supporting the theory that tau behaves in a prion-like manner, came from human post-mortem studies [99–101]. Results of a cross-sectional study of multiple brains of patients at varying stages of clinical progression showed that tau follows a stereotypical pattern of spread, which can be classified into different stages. This pattern starts initially at the entorhinal regions and locus coeruleus (stages I–II), progressing to encompass large portions of the neo-cortex (stages V–VI) (Fig. 3d) [97, 100, 101]. Named after the authors of the work, Heiko and Eva Braak, this progression and spread of tau throughout the brain was called the Braak staging system [97, 100]. Despite more recent cross-sectional and imaging studies arguing that tau spread might not be as orderly and spatially restricted as previously proposed [94], the Braak staging system is still the most accepted concept within the field, and serves as the foundation for many studies looking at propagation mechanisms in tauopathies.

Based on this initial hypothesis, more evidence in support of tau’s prion-like propagation came after the studies by Braak & Braak in 1991, using an* in vitro* system [22]. The research showed that, in cultured cell lines, extracellular tau aggregates can transmit their misfolded state from the outside to the inside of a cell, demonstrating that cells treated with tau aggregates can internalise this protein, leading to intracellular tau fibrilization [22]. Importantly, the authors also showed that aggregated intracellular tau transferred between co-cultured cells, further suggesting that the propagation of misfolded species through the brain would reflect a combination of neuronal proximity and perhaps connectivity [22]. Consistent with this concept, a follow-up study further demonstrated that misfolded preformed tau fibrils could spontaneously be up-taken by cells, proposing the likely cellular mechanism underlying this phenomenon to be endocytosis [102]. These studies were not only crucial to support the idea that tau can spread to neighbouring cells and across the neuronal circuitry, but also made ground-breaking steps in helping to identify the mechanisms of tau seeding and spread. Furthermore, the proposed mechanism of tau uptake implies that downregulation of endocytosis could be a potential mechanism to tackle the spread of tau in the brain and therefore a therapeutic target for tauopathies.

Soon after the proposal of recruitment and propagation of tau in cultured cells, the first evidence that tau could propagate in a more physiological system came to light. Clavaguera and colleagues demonstrated that injection of brain extract from mutant tau-expressing mice into the brains of transgenic wild-type-tau-expressing animals, induced assembly of tau into filaments [19]. Importantly, the study also showed that the spread of pathology started at the original site of injection, subsequently reaching neighbouring brain regions over time. The study postulated that the formation of tau aggregates in a single brain cell and their subsequent translocation and spread may occur at the origin of sporadic tauopathies. Furthermore, the authors speculated that similar to tauopathies, other neurodegenerative diseases such as PD may have a similar mechanism of prion-like spread [19].

Soon enough, the connectome hypothesis of transmission of pathological tau was validated *in vivo* [103–107], giving further evidence of the cell-to-cell transmission and seed aggregation pathway of tau. However, these studies were conducted mostly in transgenic animals, where mutations were artificially enhancing the fibrillation of tau protein *in vivo*. Added to this, the vast majority of tauopathies are sporadic in nature, raising the question as to the relevance of such models to better understand the underlying mechanisms of tauopathies. To address these points, a study looked into the spread of tau fibrils when injected into non-transgenic animals [93]. The authors demonstrated that a single intrahippocampal injection of insoluble tau fibrils purified from human AD brains, into non-transgenic mice, led to abundant propagation of tau pathology [93]. Their results showed that the pattern of tauopathy in these animals started from the site of injection and spread to its anatomically connected regions, in an extremely rapid fashion, occurring as early as 3 months post-injection [93]. Further, given the existence of different isoforms of tau, the study also investigated and compared the capacity of seeding of distinct tau conformational features and demonstrated that the seeding activity of human brain-derived tau fibrils was much higher than that of synthetic tau fibrils, which are largely used for the study of this protein [93]. Since these initial studies in non-transgenic mice, work has continued aiming to further characterise the patterns of spread. Recently, Cornblath and colleagues injected tau from AD brains into the brains of non-transgenic mice, and showed with network modelling analysis that diffusion through the connectome is the best predictor for tau pathology pattern, corroborating previous findings from others in the field [108]. This study also showed that retrograde, rather than anterograde spread, seems to dominate the spread of tau in the mouse brain [108].

Examination of tau propagation has also been further pursued, but this time investigating the seeding efficiency of different human-derived tau species. Narasimhan and colleagues showed that, like their previously published results, tau extracted from post-mortem brains of patients with different tauopathies, injected into different brain regions of wild-type mice, could induce endogenous mouse tau aggregation and propagation beyond the site of injection [109]. Interestingly, these follow-up findings showed that tau extracted from different human conditions, i.e. AD, progressive supranuclear palsy, and cortical basal degeneration, seeded with different potencies and resulted in different cell-type specificity of tau aggregate transmission [109]. Importantly, the authors also showed that what determined the pattern of pathological spread was the neuronal connectome, rather than the strain of tau itself [109]. These findings provided further evidence in support of the connectome propagation theory in tauopathies, presented a new relevant model system to study the mechanisms of sporadic tauopathies, and shed light on the physiological relevance of the currently available mouse models used to study disease processes in the field. However, given the limitations of some of these models and their translatability to the clinic, it would be prudent to verify how similar and relevant these studies are for patients with these tauopathic disorders. Although a recent work studying the seeding efficiency of α-synuclein in non-human primates has been published [110], as we will discuss later, to date tau propagation is yet to be demonstrated in higher species. Furthermore, despite some recent evidence coming from post-mortem brains of patients suggesting that tau seeding may also occur in humans [111], further studies to confirm whether tau pathology can also be ‘transmitted’ and seeded in the human brain is necessary. As such, in recent years computational modelling studies have started to appear which attempt to simulate tau’s spread throughout the human brain connectome [112], some crucially incorporating tau-PET tracer data in an attempt to validate and substantiate the connectome hypothesis of tau transmission [113, 114], or even predict future tau burden in patients [115]. Given the obvious absence of any experimental seeding data in the human brain, the aforementioned studies will be vital in order to define the translatability of those findings from pre-clinical animal experiments.

### Glymphatic clearance of tau

Is the glymphatic system involved in the clearance of extracellular tau protein which is prone to propagation in the brain? As discussed above, tau is a neuronal protein, expressed intracellularly, but there is substantial evidence to suggest that it can be secreted and taken up by both neurons and glia [116, 117]. In addition, it is thought that tau secretion to the extracellular space plays an important role in the development and spread of intracellular tau pathology [8, 48, 118, 119] (Fig. 2). Given this evidence, it could be hypothesised that the glymphatic system plays a role in clearing away the secreted extracellular tau that otherwise would remain and become prone to uptake by neighbouring cells. Indeed, in the context of traumatic brain injury (TBI), which is an established risk factor for the early development of dementia and aggregation of tau protein, it has been shown that the glymphatic system is associated with the brain’s vulnerability to neurodegeneration [48]. It was demonstrated that TBI in mice leads to decreased glymphatic influx and impaired clearance of the brain’s interstitium, which can last up to a month post-injury, promoting tau aggregation, neurodegeneration and persistent neuroinflammation in the post-traumatic brain [48]. Furthermore, it has been shown that loss of function of AQP4 impairs the clearance of interstitial solutes in the mouse brain, including the clearance of tau along the glymphatic pathway, resulting in intracellular tau aggregation and neurodegeneration, and exacerbated neurocognitive function after TBI [48].

In the context of tauopathy, we recently examined the relationship between glymphatic inflow and clearance of tau from the cortex of a transgenic mouse model of frontotemporal dementia [50]. We demonstrated that in the transgenic mouse brain, glymphatic function was impaired and the level of AQP4 polarisation to astrocytic endfeet was similarly reduced. We also showed that the degree of cortical tau deposition negatively corresponded to the level of glymphatic function in the healthy mouse cortex, alluding to the inverse relationship between the extent of glymphatic function and the degree of tau deposition in the rodent brain. We went on to show that pharmacological blockage of AQP4 resulted in the disruptions of glymphatic inflow and clearance of tau from the brain [50], strengthening the link between AQP4, glymphatic function and tau clearance. Together, these data give strong evidence to suggest that reducing the glymphatic clearance of tau may potentiate the neurodegenerative disease process, perhaps by encouraging the cell-to-cell propagation of tangle-susceptible tau protein in the brain. These results denote that AQP4 could be a powerful target for therapy in the context of tauopathic dementias.

## α-Synuclein

α-Synuclein is a cytosolic neuronal protein, which is expressed throughout the CNS. This protein has received considerable focus in the neurodegenerative disease field as it is the main component of Lewy bodies (LBs) and Lewy neurites (LNs), the main pathological hallmarks of PD [120, 121]. α-Synuclein itself is a member of the synuclein protein family, encoded by the *SNCA* gene. It comprises 1% of the total nervous system cytosolic protein and is located at the presynaptic site of neurons [122, 123]. Studies demonstrate that one of the main physiological roles of α-synuclein is to regulate synaptic neurotransmitter vesicle trafficking, by both increasing the internal Ca^2+^ release which is necessary for exocytosis and directly interacting with members of the SNARE family [120, 121]. In addition, α-synuclein is also found in different organelles of most neurons, including mitochondria, endoplasmic reticulum, Golgi apparatus, in the endolysosomal system as well as in the nuclei—although the physiological functions associated with the protein in these organelles are only partially understood [124, 125]. Given its abundance, ubiquitous presence, and interaction with numerous organelles across the cytosol, α-synuclein is regarded as a central protein for normal neuronal function, being involved in and potentially regulating many essential cellular processes.

α-Synuclein can accumulate and become cytotoxic. Inherently, α-synuclein is a 140-amino-acid soluble monomer, divided into three domains: an N-terminal domain, a non-amyloid-component (NAC) region, and a C-terminal domain [126–128] (Fig. 4b). Interestingly, α-synuclein in solution does not have a defined secondary structure [120, 129]; however, it assumes an alpha-helical conformation when bound to phospholipid membranes [130, 131]. Despite being considered an intrinsically disordered protein, it has a unique conformational plasticity which allows it to adopt a range of dynamic structures, depending on its location, environment and interactions—a characteristic potentially related to its broad physiological functions [120, 129]. It is hypothesised that while α-synuclein can be found predominantly as a monomer, it can also form stable multimeric conformations and adopt different structures under specific conditions or intracellular interactions [132]. These alternative conformations mean that α-synuclein can undergo oligomerisation/multimerisation processes which, under pathological conditions, can lead to the formation of β-sheet structured aggregates [133]. Notably, the process of misfolding, aggregation and oligomerisation gives this protein its uncommon ability to recruit additional monomers to form fibrils which are insoluble, and can therefore accumulate inside the cell [133, 134]. Importantly, these α-synuclein fibrillar species are known to be the main components found in LBs and LNs, which are usually associated with neuropathic diseases. Collectively, the group of human neurodegenerative disorders displaying altered α-synuclein in the nervous system are termed α-synucleinopathies, and include clinical entities such as PD, PD with dementia, dementia with LBs, and multiple system atrophy (MSA) [135, 136].

Intracellular aggregation of α-synuclein can be a product of different factors. It is well documented that *SNCA* gene duplication, triplication and several dominant mutations, including A30P, A53T and E46K mutations, are linked to synucleinopathies [121, 137–139]. Indeed, an extensive number of studies using* in vitro* systems and *in vivo* models have demonstrated that when overexpressed or mutated, α-synuclein is neurotoxic, and this neurotoxicity correlates with α-synuclein aggregation, therefore linking α-synuclein expression levels with the development of these diseases [137, 138, 140]. In addition to the gene/mutation expression, micro-environmental factors also play a key role in the general biology of this protein. Physical and chemical factors, such as temperature and pH variations, and the presence of other proteins or biomolecules, may affect α-synuclein aggregation processes [141]. Moreover, post-translational modifications can also interfere with the aggregation of this protein and much evidence suggests that modifications such as extensive phosphorylation as well as ubiquitination, are observed in pathological events and found in LBs [141, 142].

Further to the above mechanisms that can lead to its accumulation, clearance mechanisms can also regulate the levels of intracellular α-synuclein. Several studies have demonstrated that α-synuclein can be degraded by the ubiquitin proteasome system or by the autophagy–lysosomal pathway, and studies show that pharmacological inhibition of either of these pathways can result in α-synuclein aggregation [126, 128, 132, 143, 144]. Interestingly, it has been proposed that α-synuclein aggregates, upon formation, can inhibit the proteasome activity, creating a vicious cycle of degradation blockade and exacerbation of aggregate formation [128]. This overview on the molecular profile of α-synuclein gives an insight into its complexity and characteristics and illustrates the challenge in developing an appropriate therapeutic intervention which targets its pathological state without disrupting its intrinsic and ubiquitous physiological role.

### α-Synuclein propagation

Although the cause of sporadic PD is not known, its progression appears to have a pattern of spread. Following the proposed stages of tauopathies previously discussed in this review, Braak and colleagues also traced the course of α-synuclein pathology in incidental and symptomatic PD cases [145]. In this unique study, the brains of over 100 α-synuclein-positive individuals were examined post-mortem [145]. The authors proposed that PD-related intraneuronal pathology and neuronal damage do not happen randomly, but appear to follow a recognizable topographical pattern in the brain [145]. According to their model, there are six main stages in the evolution of the pathology, starting at the lower brainstem in the dorsal motor nucleus of the vagus nerve (DMVN) and anterior olfactory structures (stage I), then affecting the midbrain and substantia nigra (stage III), and eventually reaching the neocortex (stage VI) (Fig. 3e) [146, 147]. This study was followed by a subsequent publication where the same authors proposed that the disorder might originate outside the CNS, caused by an unknown pathogen that is capable of passing the mucosal barrier of the gastrointestinal tract and entering the CNS along the projections of the vagus nerve [146]. Led by observations that PD patients can experience several gastrointestinal dysfunctions, in addition to studies reporting the presence of LBs and LNs in the enteric nervous system (ENS) as well as in the vagus nucleus, Braak and colleagues put forward the hypothesis that α-synuclein can behave and propagate in a prion-like manner, spreading from the ENS to the DMVN *via* vagal pathways; the so-called gut-brain hypothesis [146, 148–150]. Their proposal postulates that α-synuclein can be transneuronally and retrogradely transported from the periphery and in this manner gain entry into the CNS, initially in the brainstem (DMVN), then reaching the substantia nigra *via* the nucleus tractus solitarius, and from there spreading to other areas of the CNS [146, 148–150]. Furthermore, the authors added to these remarks and proposed that the neurotropic pathogen could also enter the brain *via* the nasal route, where a nasal infection would give direct access to the olfactory nerve, transporting the pathogen in an anterograde direction, and allowing its invasion and progression into the temporal lobe [148]. Independent of the ‘infection route’, the observations from the Braak studies of sporadic PD highlight that the development of increasing degrees of Lewy pathology not only follows a predictable topographical distribution, but most importantly the predicted areas are anatomically interconnected, leading to the suggestion of a connectome hypothesis of α-synuclein propagation, similar to that described in the tau propagation field. These remarkable observations were the basis for the identification of mechanisms at the molecular and cellular levels that we know of today, which show that physical contacts, i.e., trans-synaptic and trans-neuronal transmission between vulnerable neurons, are central in α-synucleinopathies [151]. Perhaps due to the absence of a robust α-synuclein PET ligand for minimally invasive assessment of α-synuclein aggregation in humans, clinical research on the spread of α-synuclein through the brain’s connectome is hampered, and research to date has focused largely on animal [152] and mathematical [153] models.

Following the proposed Braak stages, early evidence suggested that α-synuclein could be found in intracellular vesicles, and when newly synthesized could be secreted from cells through an exocytotic pathway [154], adding to the hypothesis that this protein could be transmitted in a prion-like manner. Several *in vitro* studies followed, demonstrating that the extracellular α-synuclein can be internalized by cells, many reporting the cytotoxic effects of extracellular α-synuclein when added to the culture medium [30, 31, 155, 156]. Importantly, later studies have indeed validated this theory, by demonstrating that α-synuclein can be up-taken from the extracellular space and also transmit between cells [21, 157]. Using an *in vitro* system, the first study of this kind showed that when neuronal cells overexpressing human α-synuclein were co-cultured with different neuronal cells, they could act as donors of α-synuclein to the other cells [21]. This *in vitro* study demonstrated that exposure to mutated cells can lead to the accumulation of transmitted α-synuclein in host cells, forming Lewy-like inclusions which could lead to neurotoxicity and apoptosis [21].

But how could some sparse extracellular manipulated molecules accumulate intracellularly in a neighbouring cell? To address this question, an elegant study by Luk and colleagues looked at the effects of monomeric and fibrillar α-synuclein in culture [157]. Although the study could not reproduce some of the observations described in previous studies (e.g. [21]), the authors demonstrated that introduction of exogenously assembled α-synuclein fibrils could recruit endogenous soluble α-synuclein protein, converting it into insoluble inclusions [157]. This study was the first indication that exogenous α-synuclein fibrils could act as a catalyser of intracellular α-synuclein, which in turn forms aggregates and structures that closely resemble LBs present in the brains of patients with synucleinopathies [157]. Importantly, the results of this study also showed that neither monomeric nor oligomeric forms of α-synuclein were capable of efficient recruitment or conversion of soluble endogenous proteins into a misfolded state, making evident the crucial importance of a molecular entity capable of corrupting the endogenous protein [157]. Together with later reports further demonstrating that exogenous α-synuclein can lead to pathological hallmarks which resemble PD *in vitro* [158, 159], these studies were pioneering in demonstrating the seeding property of α-synuclein and its intrinsic spreadable, prion-like nature, in a dish.

Cell-to-cell transmission of pathologic α-synuclein also occurs *in vivo*. Evidence in support of such a mechanism came from post-mortem studies of patients who received striatal grafts of foetal human midbrain neurons. Upon post-mortem examination, it was reported that patients who had long-term survival of transplanted foetal mesencephalic dopaminergic neurons (> 10 years) developed α-synuclein–positive LBs in grafted neurons, suggestive of propagation from neighbouring α-synuclein-laden host cells [160–163]. These data, along with the strong evidence for the seeding nature of α-synuclein *in vitro*, led to revolutionary studies investigating the effects of intracerebral inoculation of α-synuclein in mice.

In an initial study, young α-synuclein-mutant transgenic mice, displaying no synucleinopathy phenotype, were given a striatal injection of brain homogenates derived from older mice exhibiting α-synuclein pathology [164]. The study showed that intracerebral inoculation of α-synuclein induced pathological propagation throughout the CNS, to regions far beyond the initial injection sites, initiating PD-like LBs/LNs across different regions of the brain as early as 30 days post-injection [164]. Importantly, the authors reported that inoculation of fibrils assembled from recombinant human α-synuclein induced identical consequences, accelerating and increasing accumulation of α-synuclein pathology in the brain, as well as dramatically reducing the survival of the animals [164]. Although these experiments were pioneering in demonstrating the prion-like cascade nature of synucleinopathies *in vivo*, it remained to be investigated whether this protein would behave in the same manner in a non-genetically manipulated model system.

To address this question, a follow-up study injected wild-type non-transgenic mice with synthetic α-synuclein fibrils and longitudinally characterised their phenotype [165]. In this innovative study, the authors showed that a single intrastriatal inoculation of α-synuclein fibrils could lead to the cell-to-cell transmission of pathologic α-synuclein and Parkinson’s-like Lewy pathology [165]. Strikingly, their histological results showed accumulation of α-synuclein in the brain from day 30 after the inoculation, spreading through anatomically interconnected regions, as well as reaching the contralateral hemisphere, although fairly modestly, after 30 days [165]. The study also highlighted that progressive accumulation of α-synuclein led to a noticeable loss of dopaminergic neurons, particularly in the substantia nigra, resulting in the development of motor deficits in the animals, resembling the classical pathology and symptoms of PD patients [165]. Further, it has since been demonstrated that, when seeded, the concentration of fibrils controls inclusion formation and neurodegeneration phenotypes *in vivo*, and that different wild-type rodent strains can show distinct phenotype outcomes when the same concentration of α-synuclein is used for seeding [166]. Although it is still unclear why inclusion burden, rate of spread and dopaminergic neurodegeneration differ, the data highlight how strain effects could potentially influence results and their interpretation in an experimental setting. Subsequent *in vivo* studies in rodents have further demonstrated the intrinsic prion-like spread of α-synuclein, including a study where homogenates from the brains of patients with LBs were used as the initial pathological seed [23, 158, 167]. Further, it has been demonstrated more recently that substantial inter-case variability in the propensity of patient-derived α-synuclein fibrils also exists [168], alluding to the complex nature of the prion-like propagation of α-synuclein in the brain.

Further to the observations in rodents, a recent study uniquely evaluated the effects of intrastriatal injection of α-synuclein fibrils in non-human primates, in a year-long longitudinal study [110]. Using non-invasive* in vivo* imaging methods, the authors showed a significant alteration in dopamine transporter binding in the brain areas immediately connected to the region injected with α-synuclein fibrils, a change that became increasingly more prominent over the course of the study [110]. Importantly, the study also showed that α-synuclein fibril injection in the brains of monkeys resulted in Lewy pathology in the substantia nigra, accompanied by a significant reduction of dopaminergic neurons in this area, as observed in early PD [110]. Collectively, these findings were pioneering in demonstrating the prion-like cascade in synucleinopathies *in vivo* and across species. Together with the early proposals by Braak, these studies solidified the concept of cell-to-cell transmission and propagation of misfolded α-synuclein, which is thought to underlie the CNS spread of LBs/LNs. Furthermore, by demonstrating that α-synuclein fibrils are sufficient to initiate PD-like pathology and to transmit the disease *in vivo*, these findings provide further evidence that this phenomenon could also occur in humans, as previously proposed.

### Glymphatic clearance of α-synuclein

Could the glymphatic system contribute to the clearance of cytotoxic protein in α-synucleinopathies? To date, studies of glymphatic clearance mechanisms in neurodegenerative diseases have largely focused on amyloid-β, but more recent studies showed that proteins prone to intracellular accumulation can also be subject to glymphatic clearance—as discussed above, in the case of tau. Indeed, mounting evidence indicates that α-synuclein can also be cleared by the glymphatic system, with the first indication coming from studies on MSA, a rare progressive neurodegenerative disease marked by aggregated α-synuclein in glia [169]. Post-mortem histological examination of brains of MSA patients revealed that phosphorylated α-synuclein accumulates at the astrocytic endfeet, suggesting the perivascular glymphatic clearance route of α-synuclein in the brain [170]. Further, injection of the MSA brain tissue-derived α-synuclein into the brains of mice induced an increase in widespread astrogliosis, but more interestingly the authors of this study observed an inverse relationship between the extent of astrocyte activation and the distance from α-synuclein inclusions [171]. Adding to this evidence, histological examination of brain tissues from PD patients showed a negative correlation between α-synuclein deposition and astrocytic AQP4 expression [172], implying a close relationship between glymphatic function and α-synuclein accumulation. These studies were the primary indication of a relationship between α-synuclein accumulation, AQP4 expression and polarisation in the brain. Furthermore, it highlighted that similar to tau, the glymphatic clearance pathway is also likely to play a role in the removal of CNS α-synuclein, and in disease progression of synucleinopathies such as PD.

New data from PD animal models further support the relationship between the glymphatic system and PD pathology progression. To investigate the possible involvement of meningeal lymphatic drainage and α-synuclein clearance, Zou and colleagues surgically blocked the deep cervical lymph nodes of young A53T mutant α-synuclein transgenic mice [51]. Their results showed that at 6 weeks following surgery, there was a dramatic decrease in meningeal lymphatic drainage as a result of drainage blockade, which resulted in a significant increase of α-synuclein deposition in the brains of these animals [51]. Of note, since publication of this mouse study, it has been shown that a similar scenario of impaired meningeal lymphatic drainage occurs in patients with idiopathic PD [173], adding even greater credence to initial experimental work in mice. The acceleration of the pathology observed in the mice was reflected by an increase in dopaminergic neuronal loss in the substantia nigra, which in turn led to severe behavioural motor impairments [51]. Interestingly, the study also reported a dramatic increase in the number of activated glial cells, which was accompanied by a substantial rise in the secretion of inflammatory factors [51]. However, in contrast to previous findings in patients that α-synuclein deposition was inversely proportional to astrocytic AQP4 expression and the distance from α-synuclein inclusions [171, 172], Zou and colleagues reported the opposite. Their data showed a striking increase in the level of expression of AQP4 and a decrease in its polarization, together with dense accumulation of AQP4 protein surrounding α-synuclein-positive cells in the midbrain of affected mice [51]. The discrepancies in the findings might be explained by differences in the stage of the pathology and/or area of brain examined, the technique used to evaluate AQP4 protein localisation, and most importantly, the species examined (mice as opposed to human tissue). For example, the proportion of AQP4 polarisation to glial endfeet itself is known to differ between humans and mice; therefore, it may be that the mouse brain reacts differently to such pathological challenges, i.e., attempts to clear α-synuclein by upregulating AQP4 in surrounding glia, a capability that may be deficient in human disease. Greater research focus is required to address such hypotheses and understand the relationship between glymphatic function and α-synuclein, but a very recent publication has gone some way to unequivocally linking the two. Cui et al. [52] showed that hemizygous AQP4 knockout mice which received a bi-lateral intrastriatal injection of α-synuclein pre-formed fibrils presented an accelerated pathologic deposition of α-synuclein, which in turn, facilitated the loss of dopaminergic neurons and consequent motor-behaviour impairments. Together, these findings further suggest that impaired glymphatic function, through decreased AQP4 expression, is capable of aggravating PD-like pathology in the brain, warranting further investigation of this pathway, or at least this water channel, as a therapeutic target in α-synucleinopathies.

Although research on the influence of the glymphatic system on α-synucleinopathy is in its infancy, it is clear that the above studies provide evidence for the involvement of the glymphatic system in the phenomenon of neuron-to-neuron propagation and clearance of α-synuclein from the brain. Undeniably, more studies aiming to elucidate the interplay between this novel clearance pathway and α-synuclein pathology will be necessary to understand what mechanisms are underlying the progression of the disease. This exciting and promising new area of research may potentially unveil new therapeutic targets for neurodegenerative α-synucleinopathies such as PD.

## Therapeutic potential of the glymphatic system in neurodegenerative diseases?

Is there an efficient way to increase the function of the glymphatic system? Growing evidence shows that there is a very simple way to optimise waste clearance in the CNS: by sleeping. Numerous studies indicate that circadian and sleep disturbances may drive and even accelerate the pathogenesis of neurodegenerative diseases [174], and some data make it evident that the glymphatic system may be one of the links between sleep and progression of neurodegeneration. Using a combination of live imaging and fluorescent tracers, a study has demonstrated that wakefulness significantly supresses the influx and circulation of CSF in the brains of mice [175]. In contrast, natural sleep or anaesthesia is associated with a 60% increase in CSF influx in the interstitial space, and therefore a striking increase in glymphatic function [175]. Beyond CSF tracers, the same study also demonstrated that amyloid-β is cleared two-fold faster in mice when they are asleep than when they are awake [175]. In addition, it has also been shown that the glymphatic system flushes more efficiently the metabolites produced by the CNS during sleep [63, 65], further showing a role of this system for brain homeostasis and highlighting its link to the sleep–wake cycle. In addition to these findings, it has been shown recently that the diurnal cycle of glymphatic activity is indeed controlled by the circadian rhythm itself [176].

Adding to the evidence above, a large number of studies suggest that melatonin, a hormone known to regulate the sleep–wake cycle, could potentially be used as a treatment for neurodegenerative diseases, including AD and PD [177]. Indeed, several pre-clinical and clinical studies have indicated that melatonin treatment can lead to a number of positive outcomes in animal models as well as in patients with neurodegenerative diseases [177]. These benefits appear to go well beyond the restoration of sleep–wake disturbance, and include a significant improvement in cognitive function in those with AD [177]. Although the mechanisms underlying the positive effect of melatonin treatment need to be investigated further, a recent study proposed that its beneficial effects are due to an improvement in the clearance of cytotoxic waste, both centrally and in the periphery, as demonstrated in a mouse model of PD [177, 178]. Together, this evidence suggests that sleep may have a restorative function, regulating brain homeostasis and facilitating the clearance of potential cytotoxic products that accumulate during neural activity at wakefulness.

When not asleep, exercise is thought by many to be the key to brain health. Prescription of exercise to those suffering from neurodegenerative diseases has not only been shown to help patients improve their cardiovascular, musculoskeletal and motor functions, but has also been linked to the prevention and reduction of disease progression [179, 180]. Evidence from pre-clinical studies indicates that physical activity leads to the secretion of neurotrophic factors in the CNS, enhancing synaptic plasticity and also helping with the maintenance of the blood–brain barrier [181]. Importantly, it is known that the glymphatic system is partially driven by the cardiorespiratory system, suggesting that brain clearance could benefit and also be a factor participating in the pro-cognitive effects of exercise [182]. To investigate whether the glymphatic system can benefit from physical activity, a study looked at the potential impact of voluntary exercise on the brain of an AD mouse model [183]. The study showed that aged mice that were allowed to voluntarily use a running wheel had a significant increase in glymphatic influx and efflux of ISF drainage, alongside a noticeable increase in the expression of AQP4 in astrocytic endfeet across different areas of the brain [183]. In addition, 6 weeks of voluntary running attenuated the inflammatory response in the brain and prevented synaptic loss in hippocampal and cortical areas [183]. Importantly, the study demonstrated that exercise led to a significant decrease of amyloid-β plaque load in the brain and improved cognitive function in a spatial memory task [183]. In agreement with these findings, a study using live imaging in young awake mice, demonstrated that exercise increased glymphatic function and CSF influx in widespread brain regions [182]. Although these are the first reports proposing the positive effects of physical activity on glymphatic function, they give compelling evidence for a link between exercise and increased glymphatic waste clearance in the CNS. Together, they highlight the benefits of exercise for brain health and support the use of physical activity to enhance brain function, particularly for those suffering with neurodegenerative diseases.

In summary, current evidence suggests that the glymphatic system can be positively upregulated simply by adjusting to a well-balanced, healthy lifestyle. In addition, it should be highlighted that in contrast to this, studies have shown that long-term high alcohol intake [184], recreational drug exposure (e.g. cocaine) [185], as well as chronic stress [186], can be extremely detrimental to the glymphatic system. Aside from the obvious health benefits of leading a healthy lifestyle, it could therefore be concluded that exercise and good sleep hygiene appear to be a valid approach to enhancing the glymphatic function, improving CSF flow and naturally boosting waste clearance from the brain, not only to prevent but also to slow down the progression of some neurodegenerative diseases.

But can the glymphatic system be pharmacologically manipulated? And does this represent a viable strategy to increase its activity for therapeutic gain? Osmotic agents, such as hypertonic saline and the drug mannitol, are widely used clinically for the treatment of acute brain injury, as these agents can enhance the flow of water throughout tissues, including in the CNS, and can therefore help to manage intracranial pressure [187–189]. Additionally, evidence from pre-clinical studies show that osmotic agents can facilitate the movement of particles through the brain’s interstitial spaces and consequently increase the efficiency of viral delivery to certain areas of the CNS [190–192], representing a promising delivery tool for the treatment of neuropathies. Based on this evidence, a recent study looked into the effects of manipulating plasma osmolarity on glymphatic function [193]. The results showed that hypertonic saline or mannitol treatment in mice led to plasma hyperosmolarity and resulted in a nearly fivefold increase in CSF influx into the brain and boosted the glymphatic function [193]. Interestingly, the study also showed that manipulation of plasma hypertonicity could overcome wakefulness-related inhibition of CSF influx and potentiate glymphatic function to levels similar to the sleeping state [193]. Importantly, the authors showed that hyperosmolar therapy reversed glymphatic impairment in an AD mouse model and could also improve the delivery of antibody therapy against amyloid-β in the brain [193].

These findings brought to light the use of hyperosmotic solutions to manage the glymphatic function, by increasing both clearance and delivery of solutes in the context of neuropathies. Incidentally, a small number of studies have also proposed the use of hyperosmotic solutions as a possible therapy for PD, due to their role as chemical chaperones [194]. These studies showed that high concentrations of mannitol can inhibit the formation of α-synuclein fibril aggregates in vitro [195, 196]. Interestingly, it has been reported that mannitol treatment can reverse some behaviour phenotypes in an *in vivo* model of PD (*D. melanogaste*r), and most importantly can also reduce accumulation of α-synuclein in the brain of a transgenic mouse model of the disease [196]. Although the authors of this work did not make a link between hyperosmotic solutions, the glymphatic system and the clearance of the extracellular accumulation of α-synuclein, it could be hypothesised that the results observed in the study were due to the enhancement of CSF influx into the brain and the improved glymphatic function. This, in turn, would result in an improved clearance of extracellular waste in the CNS. A similar study has recently been taken to the clinic as a Phase II trial, carried out to investigate the effects of mannitol as a disease-modifying therapeutic in PD patients [197]. If successful, the outcome of this study will represent a significant breakthrough for the treatment of PD and highlight the potential of such a therapy for other neurodegenerative diseases alike. Despite being promising, additional studies investigating whether the glymphatic system plays a central role in the findings discussed above are crucial.

As has been reviewed above, there is extensive evidence to suggest that the glymphatic system has therapeutic potential in neurodegenerative diseases; however, it is worth highlighting once more the infancy of this field of brain fluid dynamics. As such, several mechanistic aspects of the system’s inner workings are yet to be fully understood, meaning that the strategic route of most therapeutic promise in neurodegenerative diseases remains unclear at present. For instance, to the best of our knowledge, there is no evidence yet whether in the case of already stabilised intracellular aggregates, the glymphatic system can act to dissolve or clear away protein aggregates. As discussed above, studies suggest that in these cases the glymphatic system would be capable of aiding clearance of exocytosed seed components, therefore slowing down propagation, but the effect on established protein accumulations remains unclear. It also remains to be determined whether there is a limit to the amount of protein the system can clear away, if this limit is therapeutically advantageous, and if so, if it can be achieved through pharmacological/behavioural intervention strategies. Furthermore, it would be important to explore if the clearance of prion-like proteins is also dependent on the stage of the disease, i.e., more advanced disease states representing a larger challenge to the glymphatic system as a whole, and whether the clearance system itself can still work as efficiently as in the healthy/initial stages of the disease. It is clear that there is still much to learn about the role of this system in the brain and its potential as a therapeutic target in neurodegenerative disease.

## Conclusions and perspectives

**Fig. 5:**
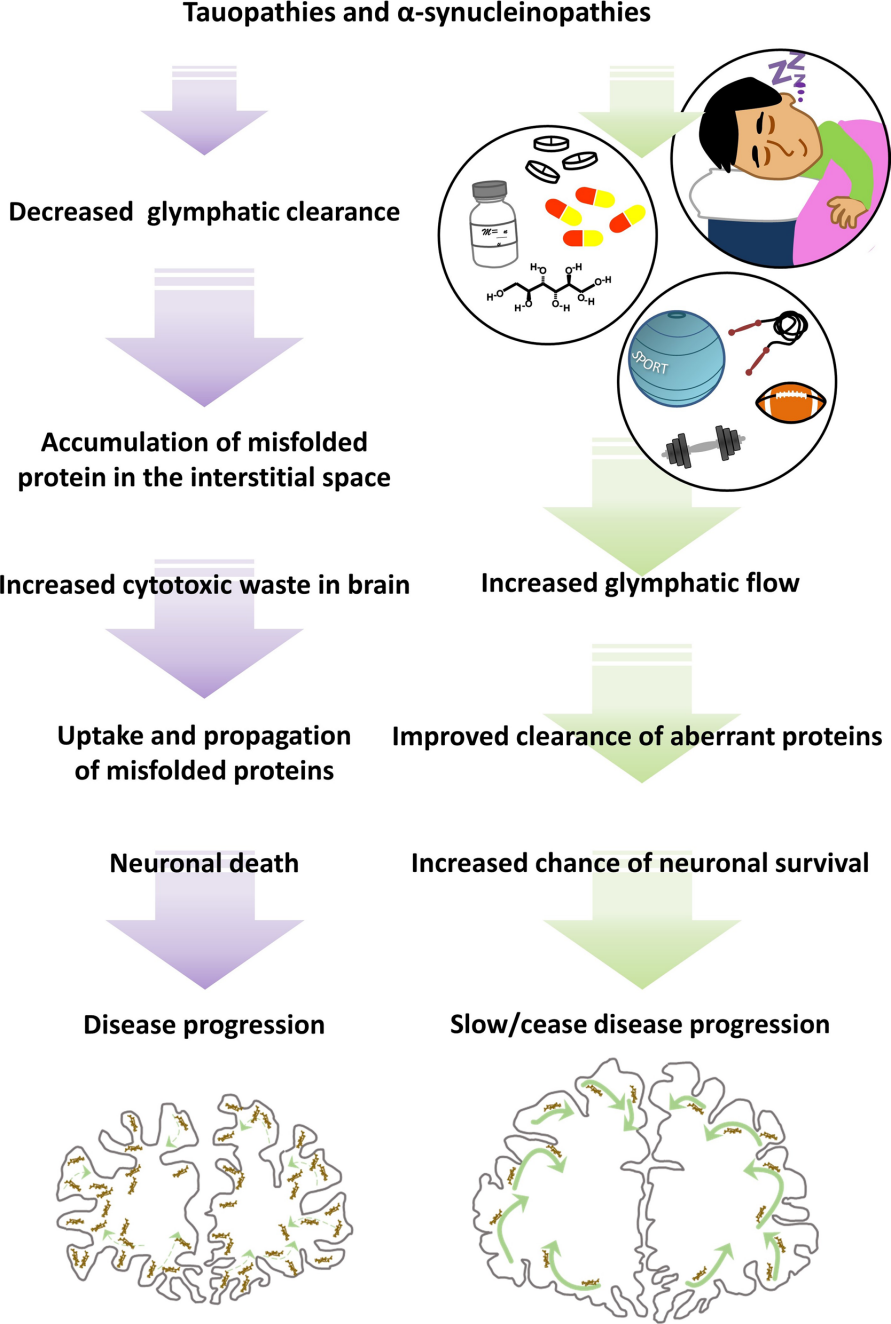
The glymphatic system in tauopathies and α-synucleinopathies. During the development of these neurodegenerative diseases (purple arrows, left), it is believed that the glymphatic function and cytotoxic protein clearance become impaired, leading to the accumulation of aberrant proteins, further seeding and propagation, and destabilisation of the neuronal network, ultimately leading to disease progression. Recent studies suggest that manipulation of the glymphatic system (green arrows, right panel)—e.g. good sleep hygiene, leading a healthy lifestyle or the administration of osmotic drugs—may have the potential to increase glymphatic function and alleviate and reduce pathogenic accumulation of intracellular cytotoxic proteins. These plausible therapeutics have the potential to increase neuronal survival and therefore delay or even prevent the progression of disease. Protein accumulation (depicted by brown aggregates) as a result of the extent of glymphatic function in the two scenarios (appropriately weighted green arrows) is represented schematically in coronal diagrams of a degenerated (left) and healthy (right) human brain cross-section in the lower section of the figure

Our increased understanding of the functional role of the glymphatic system in the clearance of proteins prone to intracellular accumulation, combined with what has been learnt in recent years about the prion-like propagation of these same proteins, has made it clear that the glymphatic system may play a substantial role in neurodegenerative diseases. The initial studies focusing on the glymphatic clearance of extracellular proteins (amyloid-β) appeared to influence the neurodegeneration research field and spurred interest in the less explored intracellular proteins tau and α-synuclein. We now know that both of these proteins can behave in a prion-like manner, can self-propagate and also spread from cell to cell throughout interconnected brain areas. Concurrent with these findings, a better understanding of the glymphatic system as a whole and its involvement in the clearance of such cytotoxic proteins in the CNS has come to light, highlighting its potentially central role in many neurological disorders. Importantly, it is becoming more evident that mechanisms of impaired brain clearance, which result in the accumulation of aberrant proteins, provide new diagnostic and therapeutic opportunities to delay or prevent clinical symptoms in diseases associated with their aggregation. Notably, mounting evidence now suggests that the glymphatic function can be manipulated. While drugs to improve the glymphatic function and its components need to be further developed and tested, based on the latest studies, we speculate that the glymphatic function can be managed by repurposing already well described drugs (Fig. 5). Furthermore, we also propose that beyond the well-known benefits, leading a healthy lifestyle may also have a positive impact on glymphatic function. Although there is much to learn about the glymphatic system’s function and its interaction with prion-like proteins, it is clear that these fast-moving fields together play a central role in neurodegenerative diseases.

## Data Availability

Not applicable.

## Abbreviations

AD: Alzheimer’s disease
AQP4: Aquaporin-4
Ca^2^^+^: Calcium ion
CNS: Central nervous system
CSF: Cerebrospinal fluid
DMVN: Dorsal motor nucleus of the vagus nerve
HSPGs: Heparan sulphate proteoglycans
ISF: Interstitial fluid
LB: Lewy body
LN: Lewy neurite
MSA: Multiple systems atrophy
NFT: Neurofibrillary tangle
NAC: Non-amyloid-component
PD: Parkinson’s disease
PET: Positron emission tomography
PrP: Prion protein
TBI: Traumatic brain injury
